# Supplementary material for: Recombinant Ehrlichia canis GP19 Protein as a Promising Vaccine Prototype Providing a Protective Immune Response in a Mouse Model
Source: Vet Sci. 2022 Jul 27;9(8):386. doi: 10.3390/vetsci9080386 (PMC9414908; doi:10.3390/vetsci9080386)
Supplement: Supplementary file 1 [file vetsci-09-00386-s001.zip › vetsci-1805658-supplementary.pdf]

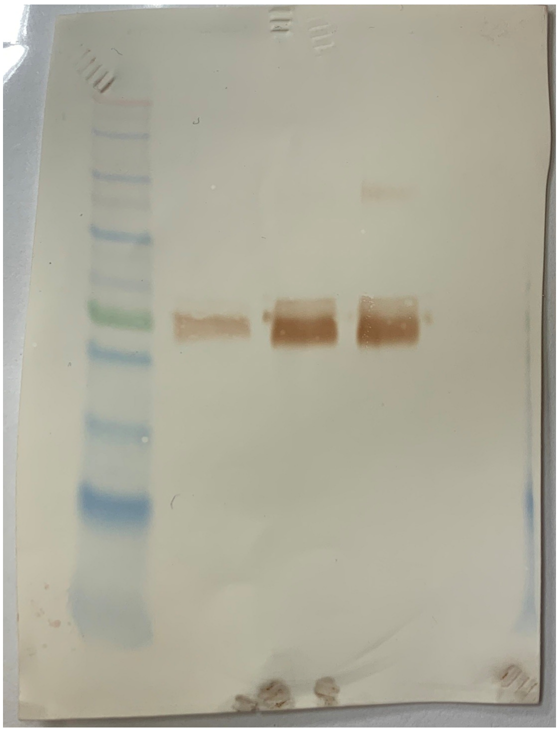

Figure S1. Recombinant protein of Ehrlichia canis (rGP19) expression affirmed with Western blot analysis.
